# Hydrocortisone Diffusion Through Synthetic Membrane, Mouse Skin, and Epiderm™ Cultured Skin

**DOI:** 10.1111/j.1753-5174.2010.00033.x

**Published:** 2011-03

**Authors:** John Mark Christensen, Monica Chang Chuong, Hang Le, Loan Pham, Ehab Bendas

**Affiliations:** Pharmaceutical Sciences, College of Pharmacy, Oregon State UniversityCorvallis, OR, USA

**Keywords:** EpiDerm™, Fick's Law, Franz Cell, Higuchi Equation, Gel, Hydrocortisone Topical Products, Permeability

## Abstract

**Objectives:**

The penetration of hydrocortisone (HC) from six topical over-the-counter products along with one prescription cream through cultured normal human-derived epidermal keratinocytes (Epiderm™), mouse skin and synthetic nylon membrane was performed as well as the effect hydrating the skin by pre-washing was explored using the Upright Franz Cell.

**Method and Results:**

Permeation of HC through EpiDerm™, mouse skin and synthetic membrane was highest with the topical HC gel formulation with prewash treatment of the membranes among seven products evaluated, 198 ± 32 µg/cm^2^, 746.32 ± 12.43 µg/cm^2^, and 1882 ± 395.18 µg/cm^2^, respectively. Pre-washing to hydrate the skin enhanced HC penetration through EpiDerm™ and mouse skin. The 24-hour HC released from topical gel with prewash treatment was 198.495 ± 32 µg/cm^2^ and 746.32 ± 12.43 µg/cm^2^ while without prewash, the 24-h HC released from topical gel was 67.2 ± 7.41 µg/cm^2^ and 653.43 ± 85.62 µg/cm^2^ though EpiDerm™ and mouse skin, respectively. HC penetration through synthetic membrane was ten times greater than through mouse skin and EpiDerm™. Generally, the shape, pattern, and rank order of HC diffusion from each commercial product was similar through each membrane.

## Introduction

Hydrocortisone (HC) has been applied topically to treat a variety of conditions due to its anti-pruritic and anti-inflammatory properties [1,2]. Several different membranes have been used to study permeation of drug including synthetic membranes [3], animal skin and human skin donated from either cadaver or plastic surgery [4–7]. Cellulose acetate membrane with 0.45 µm pore size was one of several synthetic membranes that have been used to determine the *in vitro* drug release profile of HC from creams, ointments, and lotions using diffusion Franz cell system [3]. Cellulose acetate with or without the wetting agent of isopropyl myristate containing 15% ethoxylated aliphatic amine had no significant effect on HC release from lotion and cream but significantly enhanced the release of HC from ointment [3]. The cumulative amount of HC released from the cream was found to be linear and directly proportional to the square root of time or followed Higuchi's diffusion controlled model. The HC release was faster from 2.5% compared to 1% creams, which in turn was faster than from 0.5% HC cream [3]. The release rate ranged between 0.61 and 2.68 µg/cm^2^/min^0.5^ for betamethsone dipropionate depending on the percentage of ethanol in the receptor medium [8]. Polysulfone synthetic membrane was used to determine the release rates from the corticosteroid ointments [9] and yielded nearly identical release rates as the mixed cellulose acetate/cellulose nitrate membrane [10]. Shah's observations were that the characteristics of the membrane do not generally contribute to release rate of HC from topical dosage forms, provided that sufficient porosity of the membrane is maintained [8]. A novel topical drug delivery vehicle of Span 20: Span 80 proniosomes showed an increase in diffusion of HC (1%) through the skin (58.29%) [11]. The HC released was linear to the square root of time.

Formulation of drug products for topical drug administration is best assessed using a membrane that is as similar as possible to human skin [12,13]. As there is limited availability of human skin and securing and using animal skin has its own drawbacks, cultured human epidermal keratinocytes could possibly assist in formulation of topical drug products. Normal human-derived epidermal keratinocytes (NHEK, EpiDerm™) cultured to form a multilayered, highly differentiated model of the human epidermis is viable alternative. NHEK has a well developed basement membrane, *in vivo*-like lipid profile and has been used *in-vitro* to assess dermal irritancy and toxicology [14,15].

As NHEK is readily available this study was performed to assess the usefulness of NHEK as a permeation barrier. Three different membranes (synthetic membrane, mouse skin and NHEK (EpiDerm™)) were used to compare the diffusion of HC from six OTC HC products and one prescription HC product. A comparison of release rates from the commercial HC products should also allow better utility and use of the topical products in topical HC therapy.

## Materials and Methods

### Reagents and Standards

Hydrocortisone USP reference standard (Lot 20011), propylparaben USP reference standard (Lot 130011) were obtained from USP (Rockville, MD). Absolute—200 Proof Ethyl alcohol USP was obtained from AAPER Alcohol and Chemical Co. (Shelbyville, KY). HPLC-grade acetonitrile and methanol were from EMD Chemicals, Inc. (Gibbstown, NJ), FD&C Blue from Allied Chemical & Dye Co. (New York, NY).

### Topical Hydrocortisone Products Studied

Six over-the-counter 1% HC products: one gel, one ointment, one lotion and three creams were evaluated, in addition to a prescription HC cream USP, 2.5% (Table 1).

**Table 1:** The studied formulations: 1 gel, 1 ointment, 1 lotion and 4 creams

Product name	Dosage form	Strength	Packaging	OTC or prescriptive	Manufacturer
Cortaid	Lotion	1%	Pump bottle	OTC	Johnson & Johnson
Cortaid	Cream	1%	Plastic tube	OTC	Johnson & Johnson
Aveeno	Cream	1%	Plastic tube	OTC	Johnson & Johnson
Cortizone-10 Plus	Cream	1%	Plastic tube	OTC	Pfizer
Cortizon-10	Ointment	1%	Plastic tube	OTC	Pfizer
Hydrocortisone	Cream	2.5%	Metal tube	Prescriptive	E. Fougera & Co
Corticool	Gel	1%	Plastic tube	OTC	Tec Labs

### Synthetic Membrane, Mouse Skin and EpiDerm™ Cultured Human Skin

Forty-seven mm diameter, 0.45-µm porosity Nylaflo nylon membrane filters, purchased from Pall Life Science (Ann Arbor, MI) served as the synthetic membrane. Mouse skin was provided as a gift from Dr. Rong Cui (Oregon State University College of Pharmacy). Immediately upon sacrificing the mice, the skin was excised. The skin was cut into approximately 35-mm diameter ovals and immediately mounted on the Franz cells for the diffusion study after the hair and the subcutaneous fat were removed. Twenty four normal human-derived epidermal keranocytes (NHEK; EpiDerm™) cell cultured inserts were obtained four separate times from MatTek (Ashland, MA). NHEK cell culture inserts were immediately mounted on the Franz cells for study within 1 hour of an arrival. While NHEK cultured cells can be refrigerated for four days and remain viable it is best to immediately use them upon arrival for best results as storage conditions may be rigorous for cells.

### Transepidemal Waterloss TEWL (g/h/m^2^)

The living tissue membranes (mouse skin and NHEK) were tested for viability by the TEWL test at time points 0 (the mouse skin was excised or NHEK arrival) and at 10 and 24 hours after the membranes were mounted on the Franz cells for the diffusion studies.

The open chamber measurements were carried out at normal room condition (20°C and 40–60% air humidity) using Multi Probe Adapter MPA and its probe- The Tewameter®TM300 (CK electronic GmbH, Mathias-Bruggen-Str.91 D-50829 Koln/Germany) in Dr. Arup K. Indra's laboratory (Department of Pharmaceutical Sciences, College of Pharmacy, Oregon State University).

### HPLC Assay Development and Validation

The HPLC assay was adapted from HC cream monograph, USP 33/NF28 (Rockville, MD). An equal amount of acetonitrile was added to the collected samples to precipitate protein prior to the addition of the internal standard, propylparaben. Filtered and degassed solution of water, methanol and acetonitrile (61:19.5:19.5) was used as the mobile phase to ensure separation of HC, internal standard and unidentified peaks for all tested HC products. The HC retention time was 22 minutes. The HPLC system with autoinjector consisted of a C_18_ column (3.9 × 150 mm, 5 µ, Waters, Milford, MA) with a precolumn (Model 590, Waters, Milford, MA), a UV-VIS wavelength detector set at 254 nm and an integrator recorder (Model 740 Data Module, Waters, Milford, MA). The injection volume was 50 µL. Flow rate of the pump was 1 mL/min. The standard curve was reproducible and exhibited linearity in the range from 0.5 to 120 µg/mL.

### In Vitro Permeation Study

Synthetic Nylaflo, mouse skin and NHEK were used as the barrier for the permeation study. The effect of pre-washing skin for 30 sec prior to applying HC products was also evaluated in order to assess the effect of hydrating skin versus non-hydrating skin with a surfactant solution on HC diffusion. Also prewashing may remove a small amount of the outer layer of the skin and affect diffusion. While thickness of mouse skin and Epiderm™ was measured, no significant differences were observed.

The Upright Franz Cell (Crown Glass, Somerville, NJ) consisted of 21 vertical cells (each with a nominal diameter of 2.2 cm orifice and a receiver capacity of approximately 14 mL internal sample receptor vessel volume with sampling port) and a water bath (Forma Scientific, Marietta, OH) used to maintain the temperature at 32 ± 1°C. The receiver chambers were filled with an aqueous solution containing 25% ethyl alcohol and were stirred at 300 rpm using a mini magnetic stirrer. The entire surfaces of the mounted membranes were in direct constant contact with the receiver medium and the medium in receiver chambers was maintained at temperature of 32 ± 1°C. All the membranes were pre-wetted in the aqueous receptor medium for 15 minutes before use. A sample specimen from each topical product was loaded onto weighing paper and approximately 1 gram weight of product cream, gel or ointment was measured. After transferring the weighed material of the topical product (the permeate) onto the center of the pre-wetted membrane, the weighing paper and permeate left on the weighing paper were measured again to determine the exact amount of permeate loaded on the membrane. During membrane diffusion study, 150 µL aliquot was taken from the sampling port at 0.5, 1.5, 3, 6, 10, 20 and 24 hours. After each sample was withdrawn, the receptor medium was replenished with 150 µL of fresh medium. A 50 µL aliquot of the collected sample was used in the HPLC assay. For each diffusion test run in the study, a minimum of three Franz diffusion cells were set up for each topical product with each membrane and each HC *in vitro* diffusion test was run in duplicate or triplicate with the gel formulation being run as many as nine times (in Tables an n of 2 or 3 represents 6 or 9 individual cells).

### Data Management

The release profile of HC was determined over 24 hours and plotted based upon total amount of drug loaded as cumulative amount of loaded drug released versus time (Fick's law) or square root of time as suggested by Higuchi [3,16–18].

Commercial HC has melting point of 212–213°C and solubility of 0.28 mg/mL in water, 15 mg/mL in alcohol, and 12.7 mg/mL in propylene glycol at 25°C. The commercial gel product was formulated as a clear solution of 10 mg/g.

For Fick's law [19],


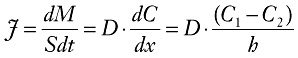
(1)


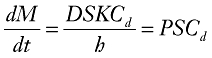
(2)


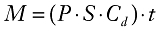
(3)


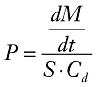
(4)

Where M is amount of drug (mg), J is flux of drug (mg/cm^2^/hr), K is distribution or partition coefficient, S is unit cross section (cm^2^) and P is permeability coefficient (cm/hr). 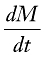
is the fitted release rate of the HC release versus time profile.

Higuchi developed an equation for the release of a drug from an ointment base and later applied it to diffusion of solid drugs dispersed in homogeneous and granular matrix systems and the amount of drug depleted per unit area of the system, Q, at time t, is given by the Higuchi equation [20]. This equation, grounded on principles of diffusion as expressed by Fick's first law of diffusion, describes the release of a drug from topical dosage forms such as gels, creams, and ointments.


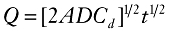
(5)


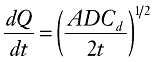
(6)


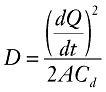
(7)

Where Q is the amount of drug depleted per unit area of the matrix at time t (mg/cm^2^), dQ/dt is the rate of drug released per unit area of exposed surface of the matrix,

D is the diffusion coefficient of the drug in the matrix (mg·mL/cm^4^/hr),
C_d_ is the solubility of the drug in the homogeneous matrix (formulation) (mg/cm^3^), C_d_ = 1 mg/mL for gel formulated as a solution within a clear gel. A is the total concentration of dissolved and undissolved of the drug in the matrix (mg/cm^3^).

After the HC release rate (µg/cm^2^/hr^1/2^) was obtained from the diffusion profile, the amount of drug released per unit area (µg/cm^2^) was plotted against the square root of time and the diffusivity or diffusion coefficient, D, was calculated from Eq. 7 to assess the release profile for HC from each product. The total quantity for each topical formulation placed on the membrane was measured and was approximately 1 g for each Franz cell well. The drug penetrating through the membranes to the receiver chamber was analyzed by the HPLC assay. The amount and the release rate over 24 hours was calculated with the assumption that the theoretical maximum amount that can diffuse into the donor chamber is 25 mg for the prescription HC cream 2.5% and 10 mg for the 1% HC topical formulations.

### Statistics Method

To compare drug release behavior of all formulations through “pre-wash” and “non-washed” skin, simple paired *t*-tests were performed at 10, 20 and 24 hours on HC diffusion rates of the topical products.

ANOVA for Multiple- linear regression method was used to compare the release rates of HC gel formulation to the other commercial formulations [21]. A 95% confidence interval band was set up for HC diffusion profile of gel formulation through each membrane. If any specific point from the amount released profile for the other reference formulations (ointments and creams) lies outside the confidence interval band of gel formulation within the last four time points (6, 10, 20, 24 hours), the HC release profile of gel formulation is considered to be significantly different from the other topical formulations.

## Results

### Dissolution Profiles with Nylaflo Synthetic Membrane Filters

**Figure 1:**
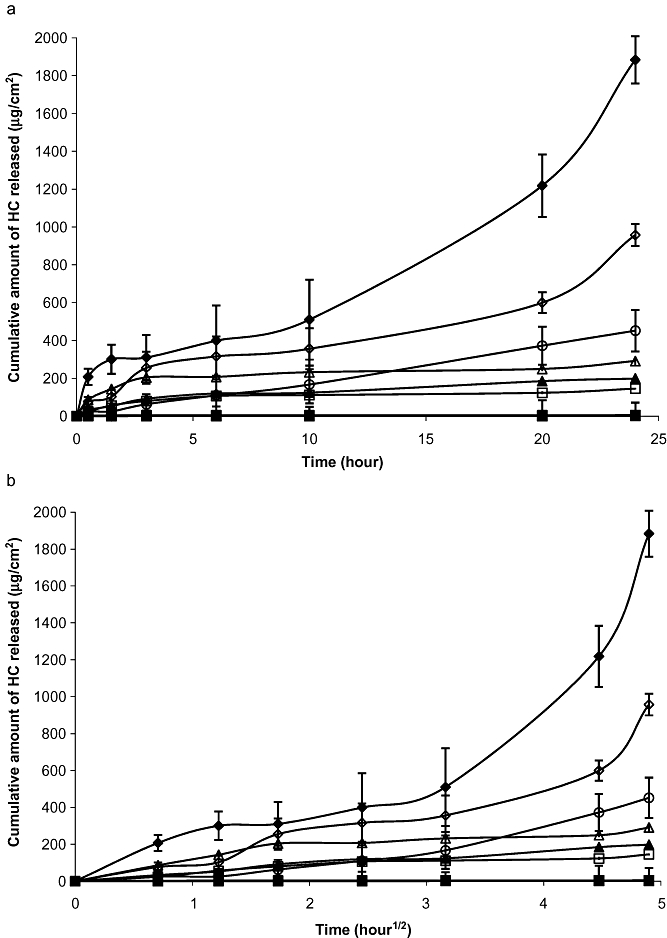
(a) Hydrocortisone diffusion profile from topical formulations over time through synthetic membrane. 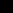 = Cortisone-10 Ointment, □ = Cortisone-10 Plus Cream; ▴ = Cortaid Cream, ♦: Corticool gel, ▵ = Aveeno Cream, ○ = Prescription HC Cream 2.5%, ◊: Cortaid lotion. (b) Diffusion profile of Hydrocortisone versus the square root of time from formulations through synthetic membrane. 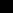 = Cortisone-10 Ointment, □ = Cortisone-10 Plus Cream; ▴ = Cortaid Cream, ♦: Corticool gel, ▵ = Aveeno Cream, ○ = Prescription HC Cream 2.5%, ◊: Cortaid lotion.

The cumulative amount (µg/cm^2^) of HC released from the commercial products through synthetic membrane was plotted against time and the square root of time (hr^1/2^) and are presented in Figure 1a and b. The shape of the HC diffusion profiles was approximately linear with respect to the square root of time except from the gel formulation. The release of HC at 24 hours was 1882.7 ± 395 µg/cm^2^ for the gel formulation compared to 145–200 µg/cm^2^ for Cortaid creams. The diffusion profile of HC from the gel was significantly greater than other HC topical products with the release of HC in 24 hours reaching 59.1% ± 3.92% which is as high as the Span 20: Span 80 proniosome 1% HC formulation (58.29%) [11]. The highest reported amount was 320 µg/cm^2^ of HC released from HC lotion and creams at 6 hours through cellulose acetate from a 2.5% lotion [3] which is comparable with the present study where 399.27 ± 58.63 µg/cm^2^ HC was released from gel formulation using Nyflon membrane.

If the formulation is water-based, HC exists mostly in suspension with a small amount dissolved in water, but the majority of the HC is in solid form. Thus, without solubility enhancer, the solubility (C_d_) of HC is 0.28 mg/mL. If C_d_ changes, the slope of the rate of drug diffusion would change proportionally to the C_d_ value. In the gel HC formulation used in this study, the presence of propylene glycol and ethanol increase the solubility of HC (solubility is 12.7 mg/mL and 15 mg/mL, respectively). The use of these solvents in the gel formulation allows all the HC to be in solution in the formulation. The C_d_ of HC in the gel is 1 mg/mL and causes an increase of the rate of diffusion through the membrane as predicted by Fick's law of diffusion.

The permeability coefficients of HC from all formulations through the synthetic membrane 0.45 mm are showed in Table 2. The permeability coefficient of HC from the gel formulation taking into consideration the high solubility of HC in the gel is 7.8^-^10^−3^ cm/hour. The increase in HC solubility accounts for most of the higher HC diffusion rate.

**Table 2:** The permeability coefficients of hydrocortisone for all formulations diffusing through the synthetic membrane 0.45 µm

Formulation	*n*	Permeability coefficient(cm/hr)	R^2^
Corticool gel*	10	7.8 * 10^−3^	0.939
Cortizone-10 plus cream	5	2.555 * 10^−3^	0.728
Cortaid cream	6	2.547 * 10^−3^	0.875
Cortaid lotion	4	5.582 * 10^−3^	0.944
Aveeno cream	4	2.253 * 10^−3^	0.740
Prescription HC cream 2.5%	7	9.336 * 10^−3^	0.939

* The permeability with solubility 1 mg/mL.

Ethyl alcohol and propylene glycol increases skin penetration of drug. The presence of ethyl alcohol and propylene glycol increased the solubility of HC in the gel formulation and their combined effects cause the permeability coefficient of HC from gel to be greater than in suspensions (creams and lotions). The gel formulation is a single phase with a high percentage of water. Hydrocortisone inside the gel moves toward the membrane according to its concentration gradient, and the concentration is maintained on the surface of the membrane keeping the drug-release rate high.

With regard to the diffusion of HC through the synthetic membrane, the gel formulation had a statistically significant higher diffusion rate compared to other formulations. The average drug release from the gel formulation over 24 hours is 1895 (95% CI 1441–1950) µg/cm^2^. The HC release profiles for the other formulations fall outside of the confidence interval of gel formulation over 24 hours. The 95% confidence interval predicted from linear regression of the drug release profiles compared to the gel provide strong evidence that drug diffusion through the membrane from the gel formulation is superior to other tested formulations.

### TEWL Results

Stable, reproducible TEWL measurements were reached. TEWL values at zero time point for all the skin samples was 3.88 ± 0.0632, or in the range of values for very healthy skin conditions (0–10). At time points 10 and 24 hours, the TEWL obtained were in the range of 15–20 which showed normal skin condition.

### Permeation through Mouse Skin

**Figure 2:**
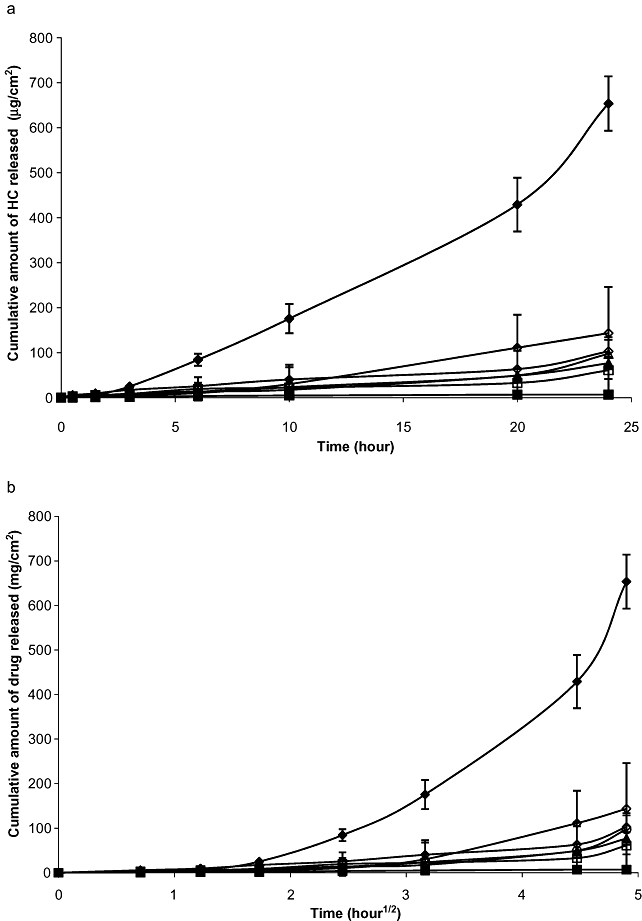
(a) The diffusion profile of hydrocortisone versus time from formulations through Non-prewashed mouse skin. 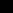 = Cortisone-10 Ointment, □ = Cortisone-10 Plus Cream; ▴ = Cortaid Cream, ♦: Corticool gel, ▵ = Aveeno Cream, ○ = Prescription HC Cream 2.5%, ◊: Cortaid lotion. (b). The diffusion of Hydrocortisone versus the square root of time profile from formulations through non-prewashed mouse skin. 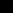 = Cortisone-10 Ointment, □ = Cortisone-10 Plus Cream; ▴ = Cortaid Cream, ♦: Corticool gel, ▵ = Aveeno Cream, ○ = Prescription HC Cream 2.5%, ◊: Cortaid lotion.

**Figure 3:**
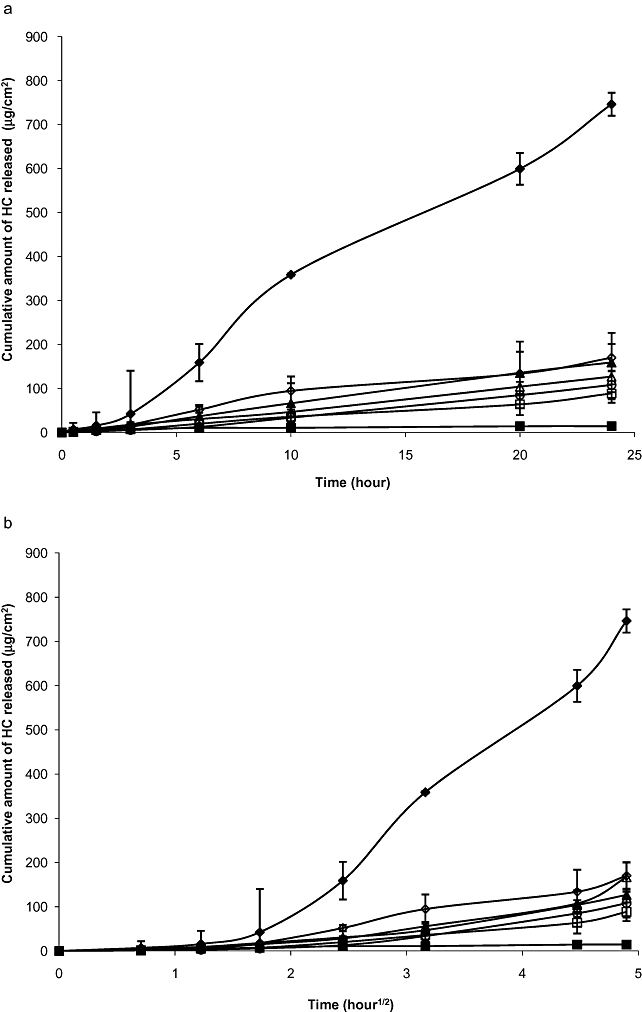
(a) Diffusion of Hydrocortisone versus time profile from formulations through the prewashed mouse skin. 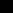 = Cortisone-10 Ointment, □ = Cortisone-10 Plus Cream; ▴ = Cortaid Cream, ♦: Corticool gel, ▵ = Aveeno Cream, ○ = Prescription HC Cream 2.5%, ◊: Cortaid lotion. (b) Diffusion of Hydrocortisone versus the square root of time profile from formulations through the prewashed mouse skin. 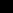 = Cortisone-10 Ointment, □ = Cortisone-10 Plus Cream; ▴ = Cortaid Cream, ♦: Corticool gel, ▵ = Aveeno Cream, ○ = Prescription HC Cream 2.5%, ◊: Cortaid lotion.

The cumulative amount of HC released from the commercial products against time and the square root of time (hr^1/2^) are presented in Figures 2 and 3: a and b. The shapes of diffusion profiles are approximately linear with respect to the square root of time with the release of HC at 24 hours being over 650 µg/cm^2^ for the gel and 50 to 160 µg/cm^2^ for other formulations. The HC permeability from the gel was calculated in a similar fashion for mouse skin as for the synthetic membrane with C_d_ = 1 mg/mL for gel, C_d_ = 1 mg/mL for the ointment and C_d_ = 0.28 mg/mL for the other formulations. The permeability coefficient of HC from the gel is close to the permeability coefficients observed for HC in the cream and the lotion. Previous studies show that alcoholic vehicles are among those reported to increase drug permeation[22]. Ethanol is primarily a lipid solvent that not only increases lipid fluidity within the intracellular space, but also extends the hydrophobic domain between the polar head groups in the stratum corneum. As a result, ethanol and other enhancers may change drug permeability. However, for HC permeation from the gel, the effect of ethanol appears to be negligible in comparison to the effect that higher HC solubility produced.

Hydrocortisone ointment's permeation profile and the release data of this formulation fit to the Higuchi model better; whereas the other formulations fit Fick's law model of diffusion. Hydrocortisone permeability coefficients of all formulations diffusing through non-prewashed mouse skin are presented in Table 3.The diffusion coefficients for HC diffusing through non-prewashed mouse skin determined using the Higuchi model are presented in Table 4.

**Table 3:** Hydrocortisone permeability coefficient and the R^2^ for linear fitting of all formulations diffusing through non-prewashed mouse skin

Formulation	*n*	Permeability coefficient(cm/hr)	R^2^
Corticool gel*	3	8.572 * 10^−3^	0.998
Cortizone-10 plus cream†	4	2.366 * 10^−3^	0.862
Cortaid cream†	5	2.138 * 10^−3^	0.961
Cortaid lotion†	3	1.326 * 10^−3^	0.987
Cortizone-10 ointment*	3	2.419 * 10^−5^	0.904
Aveeno cream†	6	1.026 * 10^3^	0.952
Prescription HC 2.5%†	6	1.264 * 10^−3^	0.916

* The permeability with solubility 1 mg/mL.

† The permeability with solubility 0.28 mg/mL.

**Table 4:** The diffusion coefficients for hydrocortisone diffusing through non-pre-washed mouse skin from commercial topical products following the Higuchi model

Formulation	*n*	Diffusion coefficient (cm^2^/hr)	R^2^
Corticool gel	3	3.465 * 10^−3^	0.969
Cortizone-10 plus cream	4	1.478 * 10^−5^	0.868
Cortaid cream	5	1.112 * 10^−5^	0.949
Cortaid lotion	3	3.415 * 10^−5^	0.977
Cortizone-10 ointment	3	8.477 * 10^−7^	0.979
Aveeno cream	6	2.685 * 10^−5^	0.832
Prescription HC 2.5%	6	3.336 * 10^−5^	0.953

#### Prewashed Mouse Skin Permeation

The HC diffusion profiles (amount permeation) for the topical HC products are displayed in Figure 3a and b. The values of HC permeability coefficients and diffusion coefficients from each topical product are presented in Tables 5 and 6.

**Table 5:** The diffusion coefficients of hydrocortisone from eight formulations diffusing through pre-washed mouse skin

Formulation	*n*	Permeability coefficient(cm/hr)	R^2^
Corticool gel*	3	3.22 * 10^−3^	0.985
Cortizone-10 plus cream †	4	1.261 * 10^−3^	0.998
Cortaid cream †	5	2.425 * 10^−3^	0.964
Cortaid lotion †	3	1.851 * 10^−3^	0.998
Cortizone-10 ointment*	3	3.194 * 10^−5^	0.904
Aveeno cream †	6	2.479 * 10^3^	0.998
Prescription HC 2.5% †	6	1.877 * 10^−3^	0.999

* The diffusion coefficient with solubility 1 mg/mL.

† The diffusion coefficient with solubility 0.28 mg/mL.

**Table 6:** The diffusion coefficients of hydrocortisone through the pre-washed mouse skin for the topical hydrocortisone formulations

Formulation	*n*	Diffusion coefficient(cm^2^/hr)	R^2^
Corticool gel	3	1.534 * 10^−3^	0.982
Cortizone-10 plus cream	4	6.590 * 10^−5^	0.909
Cortaid cream	5	9.669 * 10^−5^	0.865
Cortaid lotion	3	6.558 * 10^−5^	0.918
Cortizone-10 ointment	3	2.850 * 10^−7^	0.934
Aveeno cream	6	2.111 * 10^−5^	0.992
Prescription HC 2.5%	6	1.439 * 10^−5^	0.982

All the formulations exhibited a significantly higher HC diffusion rates through the pre-washed mouse skin compared to the non-prewashed mouse skin (*t*-tests, *P* < 0.01). As mentioned previously, hydration of the skin usually results in an increase in the drug diffusion rate through the skin and water acts as a natural penetration enhancer. An expected higher permeation of HC occurs with hydrated mouse skin.

### HC Permeation through NHEK (EpiDerm™)

**Figure 4:**
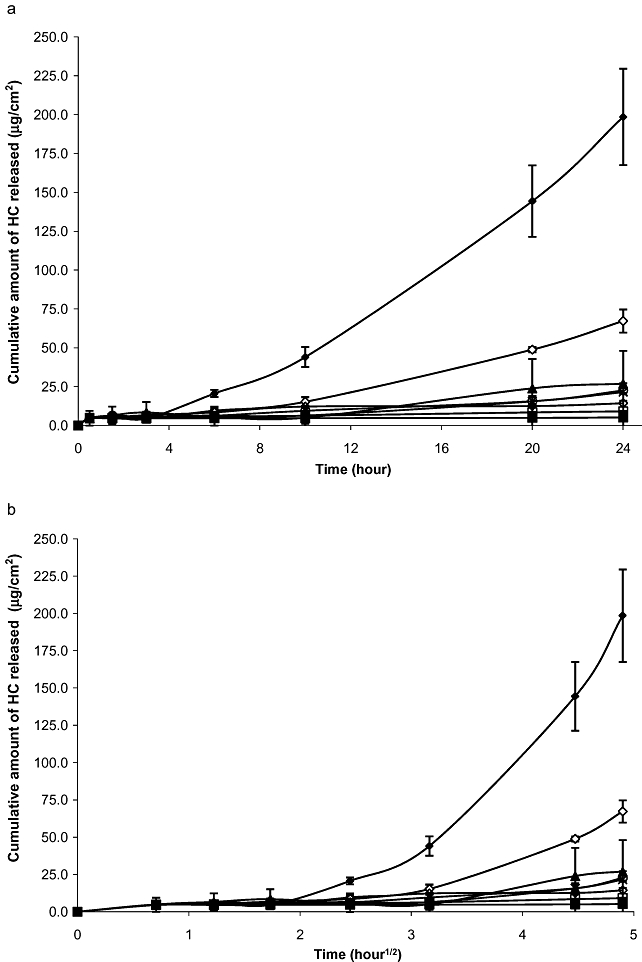
(a) Diffusion of Hydrocortisone versus time profile from formulations through NHEK (Epiderm™) over 24 hours. 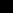 = Cortisone-10 Ointment, □ = Cortisone-10 Plus Cream; ▴ = Cortaid Cream, ♦: Corticool gel, ▵ = Aveeno Cream, ○ = Prescription HC Cream 2.5%, ◊: Cortaid lotion. (b). Diffusion of Hydrocortisone versus the square root of time profile from formulations through NHEK (Epiderm™) over 24 hours. 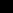 = Cortisone-10 Ointment, □ = Cortisone-10 Plus Cream; ▴ = Cortaid Cream, ♦: Corticool gel, ▵ = Aveeno Cream, ○ = Prescription HC Cream 2.5%, ◊: Cortaid lotion.

Hydrocortisone diffusion profiles from seven formulations through an Epiderm™ over 24 hours are presented in Figure 4a. The HC diffusion profiles from seven formulations plotted against the square root of time through Epiderm™ over 24 hours are presented in Figure 4b.

The diffusion profiles for HC was approximately linear with respect to the square root of time with the release of HC at 24 hours ranging from 5 to 52 µg/cm^2^ except for HC release from the gel formulation. Both diffusion profiles of HC permeation from the gel, with and without pretreatment wash, increased significantly after ten hours. The 24 hours HC release from gel was 67.20 ± 7.41 µg/cm^2^ without pretreatment and 198.46 ± 32 µg/cm^2^ with pretreatment wash.

The permeability coefficients of HC from seven topical formulations through NHEK and the diffusion coefficients of HC from seven formulations using the Higuchi model are presented in Tables 7 and 8. The permeability and diffusion coefficients for HC from gel were greater than other formulations. This can partially be explained by HC solubility in the products. Hydrocortisone in the gel is totally in solution while being in suspension in other formulations. Hydrocortisone permeability and diffusion-rate values for the gel formulation were observed to be higher through the prewashed mouse skin in comparison with non-prewashed mouse skin (Table 9).

**Table 7:** The permeability coefficients of hydrocortisone from topical formulations diffusing through NHEK (Epiderm™)

Formulation	*n*	Permeability coefficient(cm/hr)	R^2^
Corticool gel w/o	3	1.012 * 10^−3^	0.955
Cortizone-10 plus cream	3	6.366 * 10^−4^	0.995
Cortaid cream	2	2.478 * 10^−4^	0.904
Cortaid lotion	3	1.432 * 10^−4^	0.880
Cortizone-10 ointment	3	2.546 * 10^−6^	0.952
Prescription HC cream 2.5%	3	2.531 * 10^−4^	0.939

**Table 8:** The diffusion coefficients of hydrocortisone for topical commercial formulations diffusing through NHEK using the Higuchi model

Formulation	*n*	Diffusion coefficients(cm^2^/hr)	R^2^
Corticool gel w/o	2	2.318 * 10^−5^	0.848
Corticool gel w/w	1	1.987 * 10^−4^	0.861
Cortizone-10 plus cream	3	5.057 * 10^−7^	0.885
Cortaid cream	2	1.373 * 10^−6^	0.791
Cortaid lotion	3	5.603 * 10^−7^	0.947
Cortizone-10 ointment	3	1.969 * 10^−9^	0.914
Prescription HC 2.5%	6	6.833 * 10^−6^	0.658

**Table 9:** The Franz cell release rates of HC through three different membranes, derived from the Higuchi equation

	Synthetic Membranemg/cm^2^/h^1/2^	Mouse Skinmg/cm^2^/h^1/2^	EpiDerm Cultured Human Skinmg/cm^2^/h^1/2^
Cortaid lotion	0.0884 (*n* = 4)	w/o wash 0.0019 (*n* = 3) wash 0.0026 (*n* = 5)	0.0019 (*n* = 3)
Cortaid cream	0.0397 (*n* = 2)	w/o wash 0.0030 (*n* = 5) wash 0.0034 (*n* = 4)	0.0032 (*n* = 2)
Cortizone-10 plus cream	0.0275 (*n* = 6)	w/o wash 0.0011 (*n* = 4) wash 0.0026 (*n* = 4)	0.00087 (*n* = 3)
HC cream USP, 2.5%	0.0473 (*n* = 7)	w/o wash 0.0031 (*n* = 6) wash 0.0039 (*n* = 6)	0.0012 (*n* = 3)
Cortiszone-10 ointment	0.000279 (*n* = 4)	w/o wash 0.000159 (*n* = 3) wash 0.0003 (*n* = 3)	0.0004 (*n* = 3)
Aveeno cream	0.050 (*n* = 4)	w/o wash 0.0015 (*n* = 3) wash 0.0034 (*n* = 3)	Not Tested
Corticool gel	0.328 (0.5–20 h)(*n* = 10)	w/o wash 0.127 (10–24 h) (*n* = 3) wash 0.203 9 (*n* = 3)	w/o wash 0.004 (0.5–10 h) (*n* = 2)0.024 (10–24 h) with wash 0.009 (0.5–10 h) (*n* = 2)0.086 (10–24 h)

## Discussion

Percutaneous absorption data obtained in human is most relevant for human exposure [12,13]. As such, care must be taken in assessing *in vitro* studies to assess the value they shed on dermatological therapy. Still *in vitro* studies are valuable as they can provide comparisons when as is included here more than one commercial product is being tested. The data presented here provides a reasonable comparison to the commercial products and sheds light on these products usefulness in therapy, an *in vivo* study with human subjects would yield the most definitive conclusion for topical therapy. Cultured human skin (NHEK) may be an alternative to use in routine testing of topical drug products for simulating topical skin permeation studies. The 2.4 cm diameter NHEK cell culture insert allows direct mounting onto the Upright Franz Cell receptor chamber. Relatively, the shapes and patterns of HC release profiles from cream, ointment and gel commercial products were similar between synthetic nylon membrane, mouse skin and NHEK cultured human skin which was in the agreement with Shah [8] regarding the type of membranes used for topical drug release studies. This suggests that drug formulation studies utilizing NHEK membrane are effective in discriminating between topical drug formulations and can be used by the drug formulator as a membrane to characterize formulation and excipient effects on topical drug product performance.

In the present study, the HC gel formulation released the highest cumulative amount of drug through synthetic Nylon membrane (µg/cm^2^), followed by mouse skin and NHEK membrane. If the C_d_ for HC solubility is 1 mg/mL instead of the 0.28 mg/mL in Eq. 7, the HC diffusion coefficient from gel is close to other formulations: two creams and one lotion. Therefore, the high rate of HC diffusion from the gel can primarily be explained by the higher HC concentration gradient produced by the increased HC solubility between the two sides of the membrane.

The ointment-diffusion profile showed two separate phases. A short initial phase lasting the first half hour displaying an initial high rate of the drug's diffusion of the HC in layers in the ointment vehicle lying near the membrane. The HC lying deeper inside the ointment matrix needs to diffuse towards the membrane's surface before being able to diffuse through the barriermembrane. However, the high partition coefficient and high viscosity of the ointment's base prevents HC from diffusing rapidly along its concentration gradient. The whole process is slower and HC's release rate decrease dramatically. The second phase with the extremely low drug release rate is the dominant phase for HC release from the ointment.

Hydrocortisone diffusion through NHEK was 2.7 times greater from the gel formulation than the other topical formulations. From ANOVA, no significant differences in HC release were noticed among cream, lotion and ointment products; however simple paired *t*-tests showed HC release from gel and ointment to be different from the creams and lotion as well as from each other. This is likely due to low diffusion of HC through ointment and HC being in solution in the gel formulation whereas the cream and lotion formulations are suspensions. Excluding the gel, the diffusion rates of HC from the creams, and lotions through the membranes are relatively consistent in this study. However, one of the limitations of the study was the small sample size and it is possible that if more replications were performed the results may differ. Hydrocortisone release profile through NHEK culture cells yields excellent results that are consistent with other membranes in providing a discriminating tool that can be used to evaluate HC permeation.
